# Pre-emptive Circulatory Support Before Emergency Tracheal Intubation: A Systematic Review

**DOI:** 10.7759/cureus.117159

**Published:** 2026-09-29

**Authors:** Ali J Ayyashi, Majd T Alhazmi, Mohammed S Majrabi, Abdulaziz A Aqdi, Mohammed A Zaeri, Abdullah A Alshamrani, Rahiq M Hakami, Maram A Alsamti, Mashael M Alharthy, Saleh A Alquraini, Hassan Habib AlKhalaf

**Affiliations:** 1 Emergency, Medicine and Critical Care, King Fahad Central Hospital, Jazan, SAU; 2 Faculty of Medicine, Jazan University, Jazan, SAU; 3 College of Medicine, King Khalid University, Abha, SAU; 4 College of Medicine, Al-Rayan Colleges, Al-Madinah Al-Munawwarah, SAU; 5 Faculty of Medicine, Tabuk University, Tabuk, SAU; 6 College of Medicine, Taif University, Taif, SAU; 7 College of Medicine, King Faisal University, Al-Ahsa, SAU

**Keywords:** circulation-first strategy, critically ill adults, emergency tracheal intubation, haemodynamic instability, intravenous fluid, peri-intubation cardiovascular collapse, pre-emptive circulatory support, prophylactic vasopressors, rapid sequence intubation, systematic review

## Abstract

Emergency tracheal intubation is associated with peri-intubation cardiovascular collapse, and pre-emptive circulatory support is frequently used to mitigate this risk despite uncertain evidence. We conducted a systematic review of randomised and comparative non-randomised studies evaluating pre-emptive circulatory support initiated before or at induction in adults undergoing emergency or urgent tracheal intubation.

Eligible interventions included prophylactic vasopressors, intravenous fluid boluses, hypertonic saline, blood products, deliberate circulation-first strategies, and multi-component care bundles. The primary outcome was peri-intubation cardiovascular collapse within 30 minutes of induction, defined according to individual study criteria. MEDLINE via PubMed, the Cochrane Central Register of Controlled Trials, Scopus, and Web of Science Core Collection were searched from inception to 30 August 2026, with additional searches of ClinicalTrials.gov and citation lists. Risk of bias (RoB) was assessed using the Cochrane Risk of Bias 2 (RoB 2) tool for randomised trials and the Risk of Bias In Non-randomized Studies of Interventions (ROBINS-I) tool for non-randomised studies.

Fourteen independent cohorts comprising two randomised trials and 12 comparative non-randomised studies, with more than 7,400 participants overall, were included. Two randomised trials evaluated a pre-emptive 500 mL crystalloid bolus and found no reduction in peri-intubation cardiovascular collapse. Observational evidence for prophylactic vasopressors was inconsistent, with substantial changes between crude and adjusted estimates and substantial concerns regarding confounding. Studies evaluating circulation-first strategies or pre-intubation blood products generally reported associations favouring earlier circulatory resuscitation, but these findings were strongly influenced by confounding and treatment selection. Multi-component care bundles were also associated with fewer peri-intubation complications, but the independent contribution of their circulatory components could not be determined.

Overall, the available evidence indicates that pre-emptive crystalloid administration does not reduce peri-intubation cardiovascular collapse, while evidence for prophylactic vasopressors, circulation-first strategies, and multi-component interventions remains insufficient to support routine use. Further randomised trials are needed, particularly among physiologically high-risk adults.

## Introduction and background

Emergency tracheal intubation is a high-risk procedure, particularly in critically ill patients with limited cardiovascular reserve [1,2]. Peri-intubation cardiovascular collapse is a common and clinically important complication and is associated with increased mortality [3,4]. Several mechanisms contribute, including sympatholysis and vasodilatation from induction agents and reduced venous return following the transition to positive-pressure ventilation [3-7]. These effects may be poorly tolerated in patients who are already dependent on compensatory sympathetic activation, intravascular volume, or vasopressor support.

Because the risk of cardiovascular collapse can often be anticipated from pre-intubation clinical and haemodynamic factors, clinicians frequently use pre-emptive circulatory support [5,8]. Strategies include prophylactic vasopressors, intravenous fluid boluses, blood products, hypertonic saline, and deliberate circulation-first approaches in selected patients, particularly those with suspected haemorrhage [9-14]. Despite their widespread use, the effectiveness of these interventions in emergency intubation remains uncertain, and contemporary guidelines provide limited or no recommendations regarding several of these strategies.

Previous evidence syntheses have primarily focused on elective anaesthesia, surrogate haemodynamic outcomes, or broader vasopressor use rather than patient-important cardiovascular collapse during emergency intubation [2,4,6]. Evidence specific to emergency tracheal intubation is therefore fragmented, particularly for prophylactic vasopressors and other forms of pre-emptive circulatory support. Observational studies are also susceptible to confounding because clinicians are more likely to administer circulatory support to patients who are already at higher risk of cardiovascular collapse.

This systematic review synthesises randomised and comparative non-randomised evidence on pre-emptive circulatory support in adults undergoing emergency or urgent tracheal intubation, with a focus on peri-intubation cardiovascular collapse and formal assessment of risk of bias (RoB) and certainty of evidence. In adults undergoing emergency or urgent tracheal intubation, we aimed to determine whether pre-emptive circulatory support initiated before or at induction, including prophylactic vasopressors, intravenous fluid boluses, hypertonic saline, blood products, or deliberate circulation-first strategies, reduces peri-intubation cardiovascular collapse compared with usual care, no pre-emptive support, placebo, or rescue-only treatment, and to assess the certainty of the available evidence.

## Review

Methods

The review was reported according to the Preferred Reporting Items for Systematic Reviews and Meta-Analyses (PRISMA) 2020 statement [15].

Eligibility Criteria

Adults aged 16 years or older undergoing emergency or urgent tracheal intubation were eligible. Eligible settings included the emergency department, intensive care unit, hospital ward, trauma bay, and prehospital or critical-care transport settings. Emergency intubation in an operating theatre or procedural suite was also eligible. Elective operating-room intubation of haemodynamically stable patients, obstetric neuraxial anaesthesia, intubation during ongoing cardiopulmonary resuscitation, and exclusively paediatric or neonatal populations were excluded.

Eligible interventions were circulatory support initiated before or simultaneously with induction and before hypotension occurred. These included prophylactic vasopressors administered as a push-dose bolus or continuous infusion, pre-emptive intravenous crystalloid or colloid, hypertonic saline, blood products, deliberate circulation-first strategies, and combinations or protocolised care bundles containing these interventions. Circulatory support administered only as rescue treatment after hypotension had developed was excluded, as were studies comparing induction agents, airway devices, or oxygenation strategies without a relevant circulatory intervention.

Eligible comparators were usual care, no pre-emptive circulatory support, placebo, rescue-only treatment, or an airway-first sequence. Randomised trials and comparative non-randomised studies of interventions were eligible. Single-arm, descriptive, and risk-prediction studies without an intervention comparison were excluded, as were studies with fewer than 10 participants per arm. We applied no restrictions on language, publication year, publication status, or country.

For overlapping cohorts, only one report was used for a given comparison. The report representing the index cohort, based on cohort size and recruitment period, was selected, and additional reports were retained for contextual information with the overlap documented.

Outcomes

The primary outcome was peri-intubation cardiovascular collapse within 30 minutes of induction. The composite included severe hypotension, sustained hypotension, new or increased rescue vasopressor requirement, rescue fluid administration, cardiac arrest, or death, according to the definitions used in eligible studies. Where studies used an established four-component cardiovascular-collapse definition, that definition was retained.

Because rescue vasopressor and rescue fluid administration could also constitute study interventions, protocolised study drugs or fluids were not counted as outcome events. Only rescue treatment administered outside the assigned intervention contributed to the outcome. Where protocolised and rescue administration could not be distinguished, this was documented and considered during risk-of-bias assessment.

Secondary outcomes included peri-intubation hypotension, cardiac arrest, mortality, lowest systolic blood pressure and mean arterial pressure, rescue vasopressor use, arrhythmia, severe hypertension, first-attempt intubation success, peri-intubation hypoxaemia, length of stay, and ventilator-free days.

Information Sources and Search Strategy

The complete database-specific search strategies, including controlled vocabulary, free-text terms, and Boolean operators, are presented in Table 1. MEDLINE via PubMed, the Cochrane Central Register of Controlled Trials (CENTRAL), Scopus, and Web of Science Core Collection were searched from inception to 30 August 2026. The search strategy comprised three concept blocks addressing tracheal intubation and airway management, emergency or critical-care settings, and pre-emptive circulatory support. No outcome or study-design filter was applied.

**Table 1:** Database-specific search strategies Searches were adapted to the controlled vocabulary and syntax of each database. The MEDLINE search used MeSH terms and title/abstract fields; CENTRAL used MeSH descriptors and title/abstract/keyword fields; Scopus used the TITLE-ABS-KEY field; and Web of Science Core Collection used the TS topic field. No outcome terms or methodological study-design filters were applied.

Database	Search strategy
MEDLINE via PubMed	("Intubation, Intratracheal"[Mesh] OR "Airway Management"[Mesh] OR tracheal intubat*[tiab] OR endotracheal intubat*[tiab] OR emergency intubat*[tiab] OR rapid sequence intubat*[tiab] OR airway management[tiab]) AND ("Emergency Service, Hospital"[Mesh] OR "Critical Illness"[Mesh] OR "Intensive Care Units"[Mesh] OR emergency[tiab] OR critically ill[tiab] OR critical care[tiab] OR intensive care[tiab] OR trauma[tiab] OR prehospital[tiab] OR "pre-hospital"[tiab]) AND ("Vasoconstrictor Agents"[Mesh] OR "Crystalloid Solutions"[Mesh] OR "Blood Component Transfusion"[Mesh] OR vasopressor*[tiab] OR norepinephrine[tiab] OR noradrenaline[tiab] OR epinephrine[tiab] OR adrenaline[tiab] OR phenylephrine[tiab] OR metaraminol[tiab] OR vasoconstrictor*[tiab] OR fluid bolus*[tiab] OR crystalloid*[tiab] OR colloid*[tiab] OR "hypertonic saline"[tiab] OR blood product*[tiab] OR transfusion*[tiab] OR "circulation first"[tiab] OR circulation-first[tiab] OR preemptive[tiab] OR pre-emptive[tiab])
Cochrane CENTRAL	(MeSH descriptor: [Intubation, Intratracheal] explode all trees OR MeSH descriptor: [Airway Management] explode all trees OR (tracheal intubat* OR endotracheal intubat* OR emergency intubat* OR rapid sequence intubat* OR airway management):ti,ab,kw) AND (MeSH descriptor: [Emergency Service, Hospital] explode all trees OR MeSH descriptor: [Critical Illness] explode all trees OR MeSH descriptor: [Intensive Care Units] explode all trees OR (emergency OR critically ill OR critical care OR intensive care OR trauma OR prehospital OR pre-hospital):ti,ab,kw) AND (MeSH descriptor: [Vasoconstrictor Agents] explode all trees OR MeSH descriptor: [Crystalloid Solutions] explode all trees OR MeSH descriptor: [Blood Component Transfusion] explode all trees OR (vasopressor* OR norepinephrine OR noradrenaline OR epinephrine OR adrenaline OR phenylephrine OR metaraminol OR vasoconstrictor* OR fluid bolus* OR crystalloid* OR colloid* OR hypertonic saline OR blood product* OR transfusion* OR circulation first OR circulation-first OR preemptive OR pre-emptive):ti,ab,kw)
Scopus	TITLE-ABS-KEY(("tracheal intubat*" OR "endotracheal intubat*" OR "emergency intubat*" OR "rapid sequence intubat*" OR "airway management") AND (emergency OR "critically ill" OR "critical care" OR "intensive care" OR trauma OR prehospital OR "pre-hospital") AND (vasopressor* OR norepinephrine OR noradrenaline OR epinephrine OR adrenaline OR phenylephrine OR metaraminol OR vasoconstrictor* OR "fluid bolus*" OR crystalloid* OR colloid* OR "hypertonic saline" OR "blood product*" OR transfusion* OR "circulation first" OR circulation-first OR preemptive OR pre-emptive))
Web of Science Core Collection	TS=(("tracheal intubat*" OR "endotracheal intubat*" OR "emergency intubat*" OR "rapid sequence intubat*" OR "airway management") AND (emergency OR "critically ill" OR "critical care" OR "intensive care" OR trauma OR prehospital OR "pre-hospital") AND (vasopressor* OR norepinephrine OR noradrenaline OR epinephrine OR adrenaline OR phenylephrine OR metaraminol OR vasoconstrictor* OR "fluid bolus*" OR crystalloid* OR colloid* OR "hypertonic saline" OR "blood product*" OR transfusion* OR "circulation first" OR circulation-first OR preemptive OR pre-emptive))

Selection Process

Titles and abstracts were independently screened by three reviewers using a piloted eligibility form. Records that could not be clearly excluded from the abstract were advanced to full-text review. Full texts were independently assessed by the same reviewers. Disagreements were resolved through discussion, with adjudication by the principal investigator when necessary. Primary reasons for exclusion were recorded using prespecified categories. No automation tool was used to make eligibility decisions.

Data Collection and Data Items

A structured extraction form was used to record study characteristics, population, intervention, comparator, intubation procedure, outcome definitions, and outcome data. Both adjusted and unadjusted estimates were extracted when available, together with the covariates included in adjusted models. Where necessary, two-by-two tables were reconstructed from published data and checked against reported estimates.

Data extraction was performed by the review team, with a second reviewer independently checking extracted outcome data against the original reports.

RoB Assessment

Randomised trials were assessed using the Cochrane Risk of Bias 2 (RoB 2) tool [16]. Non-randomised studies were assessed using the Risk of Bias In Non-randomized Studies of Interventions (ROBINS-I) tool [17].

Because confounding by indication was considered a major threat to validity, particular attention was given to baseline blood pressure or shock index, pre-existing vasopressor use, illness severity, indication for intubation, and induction agent. The overall RoB and the specific influence of confounding were considered when interpreting study findings.

Synthesis Methods

Because of substantial clinical and methodological differences between interventions, populations, study designs, and outcome definitions, findings were synthesised narratively rather than quantitatively pooled.

Studies were organised into four prespecified strata: randomised trials of pre-emptive fluid; comparative non-randomised studies of prophylactic vasopressors; comparative non-randomised studies of circulation-first strategies and pre-intubation blood products; and studies of multi-component care bundles. These strata were considered separately and were not combined into an overall treatment effect.

For each study, the direction and magnitude of the reported association were summarised, with adjusted estimates prioritised for non-randomised studies when available. Crude and adjusted estimates were presented separately when both were reported, particularly when adjustment materially changed the direction or magnitude of the association. Differences in intervention definitions, comparator groups, outcome definitions, and RoB were considered when interpreting consistency across studies.

Results

Study Selection

Fourteen studies representing 14 independent cohorts were included in the systematic review. The study selection process is presented in Figure 1. Three groups of reports described overlapping cohorts. For each overlap, only the index cohort was used for the systematic review: the larger trauma cohort was used rather than its isolated traumatic brain injury subset [3]; the larger critical-care transport cohort was used rather than its trauma subset [4]; and the multicentre circulation-first cohort was used rather than the overlapping single-centre report [5]. One potentially eligible study identified through citation searching could not be retrieved and therefore could not be assessed for eligibility.

**Figure 1:**
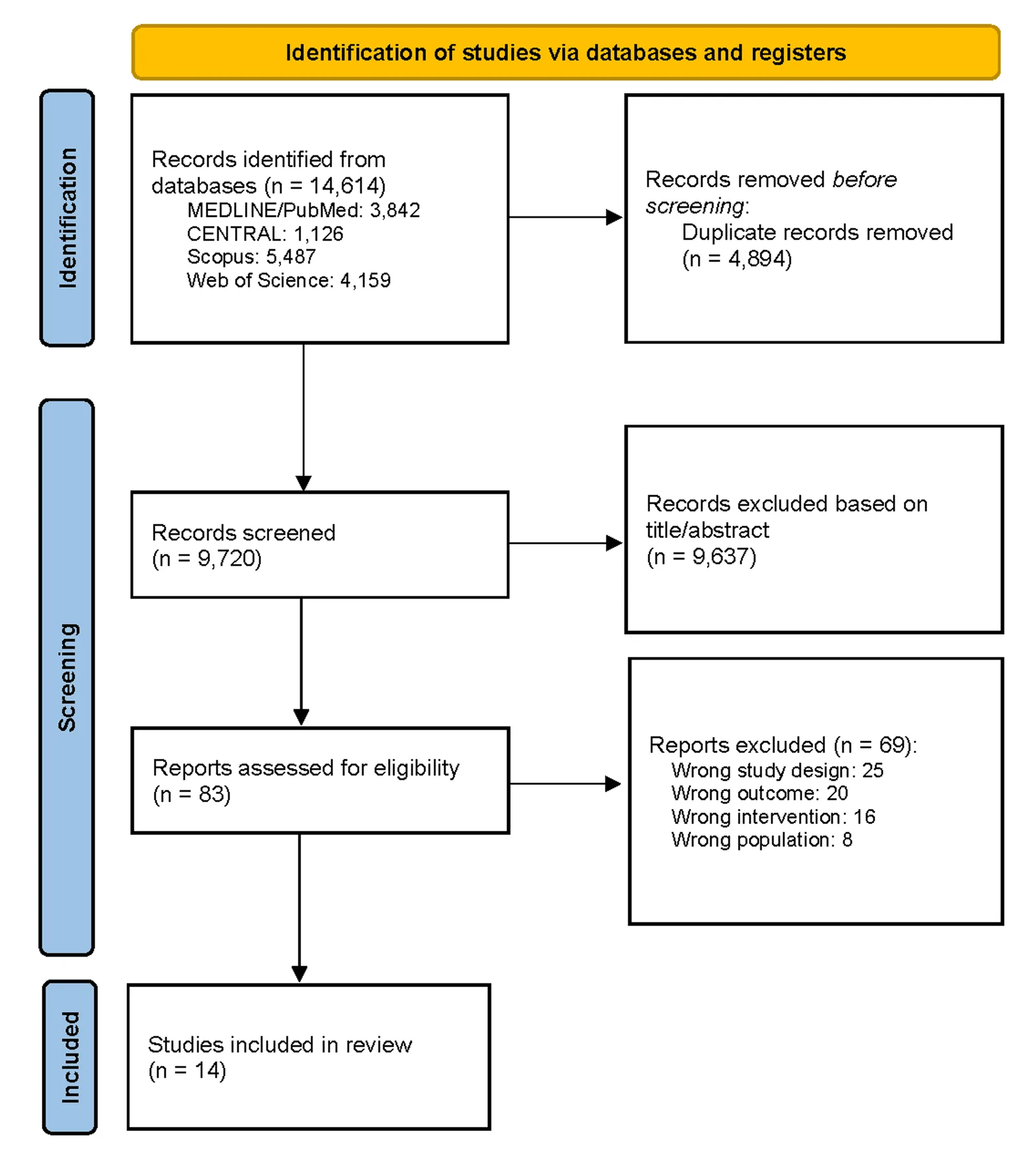
PRISMA flow diagram depicting the study selection process for the systematic review Preferred reporting items for systematic reviews and meta-analyses (PRISMA) flow diagram detailing the study selection process for this systematic review. Following the identification of records through database searching and other sources, duplicates were removed, and the remaining records were screened. Full-text articles were assessed for eligibility, with exclusions documented along with reasons. Studies meeting the inclusion criteria were included in the final synthesis.

Characteristics of Included Studies

The 14 cohorts comprised two randomised trials and 12 comparative non-randomised studies, with more than 7,400 participants overall. Studies were conducted primarily in intensive care and trauma settings, with additional cohorts from critical-care transport, emergency and mixed hospital settings, and a military surgical facility (Table 2).

**Table 2:** Characteristics and outcomes of included studies Characteristics, interventions, comparators, primary outcomes, and reported results of the 14 independent cohorts included in the systematic review. Results are presented as reported in the original studies; effect estimates are shown with 95% confidence intervals where available. RCT, randomised controlled trial; ICU, intensive care unit; PIH, post-intubation hypotension; RR, risk ratio; OR, odds ratio; IPTW, inverse probability of treatment weighting; CAB, circulation-airway-breathing; ABC, airway-breathing-circulation; HR, hazard ratio. The two fluid trials evaluated a 500-mL crystalloid bolus administered before induction. The observational studies evaluated prophylactic vasopressors, pre-intubation transfusion or circulation-first strategies, or multi-component intubation bundles. Because the bundle studies evaluated multiple simultaneous interventions, their results cannot be attributed specifically to the circulatory component.

Study	Design / setting	n	Intervention vs comparator	Primary outcome	Actual result
Janz et al. [1] – PrePARE	RCT; ICU, USA	337	500 mL crystalloid vs no bolus	Cardiovascular collapse	33/168 (19.6%) vs 31/169 (18.3%); RR 1.07 (0.69–1.67)
Russell et al. [2] – PREPARE II	RCT; 11 ICUs, USA	1065	500 mL crystalloid vs no bolus	Cardiovascular collapse	113/538 (21.0%) vs 96/527 (18.2%); RR 1.15 (0.90–1.47)
Anand et al. [3]	Prospective cohort; trauma bay, USA	708	Pre-intubation vasopressor vs none	Post-intubation hypotension	435/708 (61.4%) developed PIH; adjusted OR for vasopressor 0.12 (0.03–0.46)
da Silveira et al. [4] – ARAMAN	Retrospective cohort; 3 ICUs, Brazil	349	Push-dose metaraminol vs none	Post-intubation hypotension	36/171 (21.1%) vs 88/178 (49.4%); OR 0.27 (0.17–0.43); adjusted OR 0.14
Russotto et al. [6] – INTUBE	Prospective cohort; 197 sites/29 countries	2760	Pre-induction vasopressor/inotrope vs none	Cardiovascular collapse	Crude OR 2.34; IPTW OR 1.33 (0.84–2.11)
Fuchita et al. [7]	Secondary analysis; multicentre USA	374 matched	Prophylactic vasopressor vs none	Peri-intubation hypotension	76/187 (41%) vs 60/187 (32%); RR 1.27 (0.97–1.66); 30-d mortality HR 1.37 (1.03–1.81)
Fjeld et al. [8]	Retrospective cohort; critical-care transport, USA	333	Pre-intubation vasopressor exposure examined	Hemodynamic collapse	97/333 (29.1%) collapse; 36/333 (10.8%) life-threatening collapse; pre-intubation shock index >1: OR 3.18 (1.15–8.74)
Ferrada et al. [9] – EAST	Prospective cohort; trauma centres	278	CAB vs ABC	24-h mortality	Adjusted OR 0.11 (0.05–0.23)
Choi et al. [5]	Retrospective matched cohort; South Korea	690; 462 matched	Transfusion before vs after intubation	Mortality	24-h: OR 0.60 (0.31–1.16); 30-d: adjusted OR 0.57 (0.33–0.99)
Emerling et al. [10]	Retrospective cohort; military trauma	153	Pre-intubation blood products vs none	Mortality	24-h mortality adjusted OR 3.8 (0.7–21.5); 30-d mortality OR 1.3 (0.4–4.7)
Ferrada et al. [11] – AAST	Retrospective multicentre cohort; USA	440	CAB vs ABC	Mortality	245 CAB vs 195 ABC; mortality 47% vs 50%; no significant difference
Jaber et al. [12]	Prospective before-after; 3 ICUs, France	244 intubations	Montpellier bundle vs usual care	Life-threatening complications	21% vs 34% (P=0.03); other complications 9% vs 21% (P=0.01)
Corl et al. [13]	Prospective before-after; 3 ICUs, USA	275	Modified Montpellier bundle vs usual care	Intubation complications	All complications 42/138 (30.4%) vs 59/137 (43.1%); −12.6%; first-pass 74.6% vs 58.4%
Ghosh et al. [14]	Before-after; ICU, India	124	Revised Montpellier bundle vs usual care	Major complications	23.8% vs 45.9% (P=0.01); profound hypotension 21.77% vs 29.51% (P=0.04)

Two randomised trials evaluated a pre-emptive 500 mL crystalloid bolus [1,2]. Five cohorts evaluated prophylactic vasopressors [3,4,6-8], four evaluated circulation-first strategies or pre-intubation blood products [5,9-11], and three evaluated multi-component intubation bundles containing a fluid or vasopressor component [12-14].

Most observational studies were not specifically designed to evaluate pre-emptive circulatory support. Outcome definitions varied substantially, including composite cardiovascular-collapse outcomes, measured hypotension, and outcomes incorporating rescue treatment. This variation limited direct comparison between studies.

RoB

Both randomised trials were judged to have some concerns overall, primarily because treatment allocation could not be blinded and the primary composite included a clinician-determined treatment component [1,2]. Among the 12 non-randomised studies, one was judged to have moderate overall RoB, seven were judged to have serious risk, and four were judged to have critical risk (Tables 3, 4). Confounding was a frequent concern, particularly because of differences in baseline haemodynamic status, illness severity, pre-existing vasopressor use, indication for intubation, and treatment selection. Additional concerns varied across studies and included participant selection, deviations from intended interventions, missing outcome data, outcome measurement, and selection of the reported result. Several studies also used outcome definitions that incorporated post-intubation rescue treatment, which raised concerns regarding outcome measurement and potential contamination between the intervention and outcome.

**Table 3:** Risk-of-bias assessment of randomised trials Risk of bias (RoB) in randomised trials was assessed using the Cochrane Risk of Bias 2 (RoB 2) tool. Domains included randomisation, deviations from intended interventions, missing outcome data, measurement of the outcome, and selection of the reported result. Judgements were classified as low risk, some concerns, or high risk. Both trials were unblinded because administration of a fluid bolus could not be masked. The “new or increased vasopressor” component of the cardiovascular collapse composite was clinician-determined, whereas the remaining components were objective.

Study	Randomisation	Deviations from intended interventions	Missing outcome data	Outcome measurement	Selective reporting	Overall
Janz et al. [1]	Low	Some concerns	Low	Some concerns	Low	Some concerns
Russell et al. [2]	Low	Some concerns	Low	Some concerns	Low	Some concerns

**Table 4:** Risk-of-bias assessment of non-randomised studies RoB in non-randomised studies was assessed using the Risk of Bias in Non-randomized Studies of Interventions (ROBINS-I) tool. Domains included confounding, selection of participants, classification of interventions, deviations from intended interventions, missing data, measurement of outcomes, and selection of the reported result. Judgements were classified as low, moderate, serious, or critical RoB. Critical concerns included adjustment for post-exposure variables, absence of adjusted estimates, substantial undocumented exposure data without imputation, absence of illness-severity measures, and outcome definitions incorporating post-intubation treatment.

Study	Confounding	Selection	Classification	Deviations	Missing data	Outcome measurement	Reported result	Overall
Anand et al. [3]	Serious	Moderate	Low	Moderate	Moderate	Moderate	Moderate	Serious
da Silveira et al. [4]	Moderate	Low	Low	Moderate	Low	Low	Moderate	Moderate
Choi and Jung [5]	Serious	Low	Low	Moderate	Low	Moderate	Moderate	Serious
Russotto et al. [6]	Moderate	Low	Low	Moderate	Moderate	Serious	Low	Serious
Fuchita et al. [7]	Serious	Moderate	Low	Moderate	Low	Serious	Serious	Serious
Fjeld et al. [8]	Serious	Moderate	Moderate	Moderate	Critical	Critical	Serious	Critical
Ferrada et al. [9]	Critical	Serious	Low	Moderate	Serious	Moderate	Moderate	Critical
Emerling et al. [10]	Serious	Moderate	Low	Moderate	Moderate	Moderate	Moderate	Serious
Ferrada et al. [11]	Critical	Serious	Low	Moderate	Moderate	Serious	Serious	Critical
Jaber et al. [12]	Serious	Moderate	Serious	Serious	Moderate	Moderate	Moderate	Serious
Corl et al. [13]	Serious	Moderate	Serious	Serious	Moderate	Moderate	Moderate	Serious
Ghosh et al. [14]	Serious	Moderate	Serious	Serious	Moderate	Moderate	Moderate	Serious

Pre-Emptive Fluid Bolus

The two randomised trials evaluated the same intervention: a 500 mL crystalloid bolus administered before induction [1,2]. Neither trial demonstrated a reduction in peri-intubation cardiovascular collapse. In the first trial, cardiovascular collapse occurred in 33 of 168 patients (19.6%) receiving fluid compared with 31 of 169 patients (18.3%) receiving no bolus (RR 1.07, 95% CI 0.69-1.67). In the second trial, collapse occurred in 113 of 538 patients (21.0%) versus 96 of 527 patients (18.2%), respectively (RR 1.15, 95% CI 0.90-1.47). The direction and magnitude of the findings were consistent across the two trials. Overall, the evidence suggests that a pre-emptive 500 mL crystalloid bolus does not meaningfully reduce peri-intubation cardiovascular collapse. Certainty of evidence was judged moderate because the randomised evidence was directly applicable but remained imprecise.

Prophylactic Vasopressors

Five independent cohorts evaluated prophylactic vasopressors [3,4,6-8]. Findings were inconsistent, with estimates ranging from substantial apparent benefit to possible harm.

The most prominent pattern was the effect of confounding adjustment. In two trauma cohorts, crude analyses suggested increased risk with prophylactic vasopressors, whereas adjustment for baseline clinical characteristics substantially shifted the estimates towards benefit [3]. In contrast, the largest international cohort found no clear association after adjustment [6]. Other cohorts in which exposed patients were substantially sicker or outcome definitions incorporated post-intubation treatment also tended to report associations favouring harm [7,8].

One multicentre cohort of septic patients evaluated prophylactic push-dose metaraminol using a measured blood-pressure outcome that did not incorporate treatment administration [4]. Prophylactic metaraminol was associated with less post-intubation hypotension, and the association remained after adjustment. However, the haemodynamic benefit did not translate into a clear mortality benefit.

Taken together, the observational evidence does not establish whether prophylactic vasopressors reduce clinically important cardiovascular collapse. The marked changes between crude and adjusted estimates and the substantial differences in outcome definitions indicate a major influence of confounding and study design. Certainty of evidence was therefore judged very low.

Circulation-First Strategies and Pre-Intubation Blood Products

Four cohorts evaluated circulation-first strategies or pre-intubation blood products [5,9-11]. All reported associations favouring earlier circulatory resuscitation in at least some outcomes, but the magnitude of benefit decreased with stronger adjustment for confounding.

The strongest apparent mortality benefit came from a study in which a post-exposure variable was included in the adjustment model, creating substantial concern for bias [9]. A propensity-matched trauma cohort reported a smaller association with 30-day mortality, while another multivariable analysis found no clear mortality benefit [5,10]. A further multicentre cohort did not report an adjusted estimate [11].

These findings suggest that the apparent benefit of circulation-first strategies may be substantially influenced by differences in baseline severity and treatment selection. Because the interventions also differed in their accompanying fluid and transfusion strategies, the independent effect of the circulatory sequence could not be determined. Certainty of evidence was very low.

Multi-Component Care Bundles

Three before-and-after studies evaluated multi-component intubation bundles that included pre-intubation fluid or vasopressor elements [12-14]. All reported reductions in peri-intubation complications after bundle implementation. However, the bundles also incorporated several other interventions, including preoxygenation, induction practices, staffing, airway preparation, and post-intubation management. The contribution of the circulatory component could therefore not be isolated. Certainty of evidence was very low.

Certainty of Evidence and Reporting

Overall certainty was moderate for the effect of pre-emptive crystalloid on peri-intubation cardiovascular collapse and very low for prophylactic vasopressors, circulation-first strategies, and multi-component care bundles. The principal limitations of the observational evidence were confounding by indication, heterogeneous intervention and outcome definitions, and outcome contamination by rescue treatment.

Formal assessment of small-study effects was not possible because the evidence strata contained too few studies. Comparison with available trial-registration information identified three completed trials of prophylactic vasopressors before emergency intubation for which results have not yet been reported.

Discussion

Principal Findings

This systematic review found moderate-certainty randomised evidence that pre-emptive crystalloid administration before emergency tracheal intubation does not reduce peri-intubation cardiovascular collapse. Evidence for prophylactic vasopressors, circulation-first strategies, and multi-component bundles was predominantly observational and very low certainty. Across these interventions, substantial variation in effect estimates and shifts between crude and adjusted analyses highlight the potential influence of confounding by indication. Overall, the available evidence does not establish a routine benefit for pre-emptive circulatory support before emergency intubation.

Implications for Practice

Routine administration of intravenous fluid solely to prevent peri-intubation cardiovascular collapse is not supported by current evidence, although fluid therapy for an independent clinical indication remains appropriate. Evidence is currently insufficient to recommend routine prophylactic vasopressors or circulation-first strategies. Their use in selected high-risk patients may remain reasonable based on clinical judgement, but apparent benefits from observational studies should be interpreted cautiously. More broadly, optimisation of haemodynamic status, careful selection and dosing of induction agents, and preparation for rapid treatment of cardiovascular deterioration remain important components of emergency airway management.

Implications for Research and Limitations

Future research should prioritise randomised trials of prophylactic vasopressors and other pre-emptive strategies in physiologically high-risk adults, using patient-important outcomes that do not incorporate the intervention itself. Observational studies should clearly define exposure timing, avoid treatment-dependent composite outcomes, and report both crude and adjusted estimates. Interpretation of this review is limited by the absence of Embase, heterogeneous outcome definitions, limited randomised evidence, and substantial RoB among several observational studies. Despite these limitations, the evidence supports avoiding routine pre-emptive circulatory interventions without stronger prospective evidence.

## Conclusions

Pre-emptive crystalloid administration before emergency tracheal intubation does not appear to reduce peri-intubation cardiovascular collapse. Evidence for prophylactic vasopressors, circulation-first strategies, and multi-component interventions remains insufficient to support routine use. Further randomised research is needed to determine whether targeted pre-emptive circulatory support benefits patients at highest risk of cardiovascular collapse.
